# Comorbidities, substance abuse, weight and age are independent risk factors for postoperative complications following operation for proximal humerus fractures: a retrospective analysis of 1109 patients

**DOI:** 10.1007/s00402-021-04022-8

**Published:** 2021-07-13

**Authors:** Ralf Henkelmann, Jan Theopold, Jonas Kitsche, Paul-Vincent Link, Meinhard Mende, Pierre Hepp

**Affiliations:** 1 Department of Orthopedics, Trauma and Plastic Surgery, University of Leipzig, Liebigstraße 20, 04103 Leipzig, Germany; 2 Centre for Clinical Trials, University of Leipzig, Härtelstraße 16–18, 04107 Leipzig, Germany

**Keywords:** Postoperative complications, Proximal humerus fractures, Epidemiology

## Abstract

**Introduction:**

This study aimed to investigate the influence of epidemiologic parameters on complications that needed operative revision of operatively treated proximal humerus fractures.

**Methods:**

We performed a retrospective single-center study in a level 1 trauma center. We included all patients with operatively treated proximal humerus fractures from January 1 2005 to December 31 2015. We characterized our cohort and subgroup using descriptive statistics. The primary outcome was postoperative complications. For this purpose, postoperative complications were defined in advance, an operative revision was necessary on a general rule. The secondary outcome was a model of the risk factors for complications created with multiple logistic regression.

**Results:**

We included 1109 patients. The average age was 67.2 years (± 16.4), and 71.4% of the fractures occurred in women. A total of 644 patients (58.1%) had between one to three comorbidities, and 27.8% had four or more. The fracture morphology was as follows: 3 part 41.8%, 4 part 26.9%, 2 part 24.3%, and dislocation fracture 6.7%. Complications occurred in 150 patients (13.5%). The number of comorbidities [odds ratio (OR) 2.85, *p* < 0.01], body weight (OR 1.15, *p* = 0.02), and substance abuse (OR 1.82, *p* = 0.04) significantly correlated with the risk of complications. We achieved a sensitivity of 48% and a specificity of 74% for the variables body weight, substance abuse, age, and comorbidities

**Conclusion:**

The epidemiologic parameters, comorbidities, substance abuse, weight, and age are independent risk factors for complications. If these factors are present, one can predict a postoperative complication requiring surgical revision with low sensitivity and moderate specificity. Therefore, concerning the high number of multi-morbid patients with proximal humerus fractures, an increased postoperative complication rate can be expected.

**Level of evidence:**

Level of evidence IV

## Background

Proximal humeral fractures have an incidence rate of 60 in 1,00,000 people and are among the most common fractures. Age and osteoporosis are known risk factors for proximal humeral fractures, with a significant increase in incidence in individuals over the age of 65. These fractures occur significantly more frequently in women than in men [1–5]. Conservative treatment is preferred for non-dislocated fractures [6, 7], whereas for dislocated fractures, surgical therapy may be performed. However, the evidence for surgical therapy is based on studies that require strict pre-selection based on the inclusion and exclusion criteria due to their study design. Therefore, the types of fractures to be investigated and the surgical options are limited and may not correspond with clinical reality [3, 6–12].

Postoperative complications such as secondary dislocations, reduction losses, or screw perforations occur in 3.2–42% of cases regardless of the surgical procedure. The main reasons cited are diverse. Bone quality osteoporosis or a primary insufficient reduction of the fracture are often cited. [6–8, 10, 13]. Descriptive registry studies with epidemiological considerations have also focused on the distribution of age, sex, fracture morphology, and surgical procedure without identifying complications and their relation to these variables [1, 14, 15]. Nevertheless, the relationship between epidemiological data—e.g., comorbidities and substance abuse—and postoperative complications and the resulting predictive value have not been explicitly investigated.

Therefore, this retrospective single-center analysis aimed to identify epidemiological risk factors for postoperative complications which needed operative revision. Furthermore, we aimed to understand the patient´s characteristics to support decision-making regarding therapeutic algorithms.

## Methods

### Study design

This retrospective single-center study included all patients with surgically treated proximal humerus fractures at a level 1 trauma center from January 1 2005 to December 31 2015. The study was approved by the responsible ethics committee (494/16-ek). The consent requirement was waived because of the retrospective nature of the study and in accordance with §34 of the Saxon hospital law. Patients were filtered according to the inclusion (proximal humerus fracture with surgical treatment at the study center) and exclusion (proximal humeral fractures with primary surgical treatment at another clinic, pathological fractures, conservatively treated fractures, and cases with incomplete data) criteria.

### Patient characteristics

In addition to the standard parameters (age, sex, etc.), comorbidities were categorized into four groups according to the number of comorbidities: none, one to three, four to five, and more than six comorbidities. Comorbidities were defined as Diabetes mellitus, arterial hypertension, coronary heart disease, heart failure, asthma, COPD, emphysema, any type of tumor disease, a second tumor case regarded as independent comorbidity, apoplexy with residuals in the history, pre-existing neurological conditions (e.g., multiple sclerosis), rheumatic diseases, organ transplantation, congenital immune defects, HIV, cirrhosis of the liver, kidney failure requiring dialysis. The variables diabetes mellitus, nicotine abuse, alcohol/drug abuse, and the intake of immunosuppressants were listed separately at the nominal scale level.

Based on the social history, we defined three groups of patients; those living alone/independently, partially dependent/ in assisted care, and completely dependent/accommodated in a nursing home. The accompanying injuries were divided into none, not relevant (hematoma, abrasions, among others.) and relevant (fracture, traumatic brain injury). Accident mechanisms were divided into low- and high-energy trauma.

### Fracture classification

Fracture morphology was classified into the following groups by the attending surgeon, based on the Neer classification, and with the available X-ray and/or computed tomography images as follows: 2 part, 2 part greater tubercle, 2 part lesser tubercle, 3 part, 3 part greater tubercle, 3 part lesser tubercle, 4 part, head-split, and dislocation fractures (2 part, 3 part, 4 part, head-split) [16].

### Postoperative complications and reoperations

Postoperative complications that required revision included the following: infection, screw perforation, implant dislocation, reduction loss/secondary dislocation, dislocation of the prosthesis, pseudarthrosis, postoperative nerve lesions, and others (e.g., hematoma requiring revision). Furthermore, all other events that required a follow-up surgery that was not directly related to the initial operation (operation procedure-independent): peri-implant fracture after a fall, periprosthetic fracture, and humeral head necrosis were recorded. Analogous to the study by Hepp et al., an evaluation of the reoperations was carried out (Table 1) for further categorization and chronological classification.

**Table 1:** Specification of a reoperation according to corresponding conditions

Group	Reasons for reoperation
1	Multiple early operations with material change and/or removal
2	Multiple early operations without removal of material/with partial removal of material/with reosteosynthesis
3	An early operation with complete change of procedure
4	An early operation with partial material change (e.g. screws)/partial/complete removal of material
5	Multiple late follow-up operations with partial or complete removal of material and/or partial removal of material (e.g. screws) or later implant change
6	Late material removal with arthrolysis
7	Late material removal without arthrolysis

### Statistical analyses

The study cohort was characterized by standard statistics that included means (standard deviations) for continuous data, and numbers (%) for categorical data. Patient groups with and without complications were compared using the *t* test for continuous variables and the chi square test without correction for cross tables. If more than 20% of the contingency table had expected frequencies below five, Fisher’s exact test was used.

The sample unit was defined as the patient. For bilateral fractures that occurred one or two times, only one was randomly selected.

The risk for complications was calculated using binary logistic regression. Odds ratios (ORs) were represented by error bar diagrams. Risk factors were analyzed using a multiple logistic regression model. Starting from the base table variables, the variables were excluded stepwise backward with the probabilities for inclusion *p*_in = 0.1 and exclusion *p*_out = 0.15. For the correct estimation of the effects, a final model with the inclusion method was created with the remaining variables and ORs that included 95% confidence intervals. The final model estimated the probability of a complication for each patient graphically compared to the actual occurrence. Furthermore, the predictive quality of this probability was shown in a receiver operating characteristic (ROC) curve and the area under the curve (AUC) was calculated.

All tests were performed on both sides of the significance level *α* = 0.05. For multiple testing, the Simes method was used. The analyses were performed using IBM SPSS Statistics version 24 and R version 3.4.1 software.

## Results

### Descriptive data

We included 1137 fractures of 1109 patients with an average age of 67.2 years (standard deviation 16.4, range 16.0–97.0 years), 13 fractures occurred bilaterally one time, 15 fractures on the contralateral side occurred two times, 71.4% of the patients were women. Four or more pre-existing conditions were present in 27.4% of the patients, 25% of whom had diabetes mellitus. A total of 82.9% of the patients lived independently or alone (Table 2). In 77.2% of the patients, there was low-energy trauma*,* of which 33.5% had an isolated injury to the proximal humerus, and 60.9% had no additional injury.

**Table 2:** descriptive data patient related

	Mean	SD	Range
Age			
Years	67, 2	16, 4	(0–97)
Height			
m	1, 7	0, 1	(1.43–2.00)
Weight			
kg	74, 3	16, 8	(0–180)
BMI			
kg/m^2^	26, 8	5, 5	(1.5–64)
	*n*		Percent
Age (years)			
≤ 40	464		41.8%
40–64	79		7.1%
65–79	423		38.1%
≥ 80	143		12.9%
Gender			
Male	317		28.6%
Female	792		71.4%
Comorbidities			
None	157		14.2%
1–3	644		58.1%
4–5	175		15.8%
≥ 6	133		12.0%
Diabetes mellitus			
Yes	273		24.6%
Immunosuppressive			
Yes	40		3.6%
Smoking			
Yes	209		18.8%
Substance abuse			
Yes	110		9.9%
Living situation			
Self-sustaining	919		82.9%
Supervised	96		8.7%
Nursing home	94		8.5%

*BMI* body mass index, *SD* standard deviation

## Fracture pattern and treatment

In our study, 3 part fractures were most common, occurring in 41.8% of patients. A detailed list of fracture morphology is displayed in Table 3.

**Table 3:** Descriptive data accident fracture-related

	*N*	Percent
Injured side		
Right	560	49.3%
Left	577	50.7%
Trauma mechanism		
Low-energy	878	77.2%
High-energy	259	22.8%
Concomitant injury of affected extremity		
None	381	33.5%
Relevant	141	12.4%
Not relevant	615	54.1%
Concomitant injury other		
None	692	60.9%
Relevant	174	15.3%
Not relevant	271	23.8%
Fracture pattern		
Unknown	4	0.4%
2part	220	19.6%
2part tub. Majus	46	4.0%
2part tub. Minus	10	0.9%
3part	34	3.0%
3part tub. Majus	419	36.8%
3part tub. Minus	22	1.9%
4part	258	22.6%
Headsplit	48	4.2%
2part luxation	36	3.1%
3part luxation	21	1.8%
4part luxation	18	1.6%
Headsplit luxation	1	0.1%
Treatment strategy		
Plate	665	58.5%
Intramedullary nail	242	21.3%
HAS	116	10.2%
RSA	63	5.5%
Screws	39	3.4%
Double plate	12	1.1%

*RSA* reversed shoulder arthroplasty, *HAS* hemi-shoulder arthroplasty

The treatment strategy was as follows: plate osteosynthesis (58.5%), intramedullary nail (21.3%), hemi-shoulder arthroplasty (HSA 10.2%), reversed shoulder arthroplasty (RSA 5.5%), screw osteosynthesis (3.4%), and double plate osteosynthesis (1.1% Table 3).

### Complications due to surgery

**Fig. 1:**
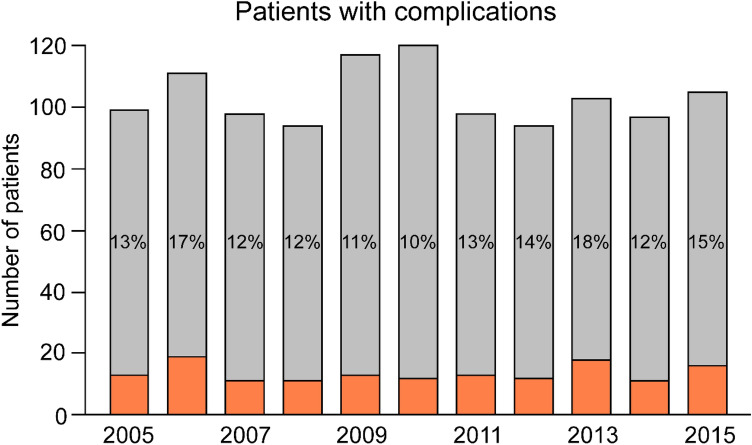
Number of operations and complications per year

There were complications in 150 cases (13.5%). Figure 1 illustrates the total number of patients and the annual complication rate. There was no significant difference between the years.

Secondary fragment dislocation and loss of reduction were the most frequent complications with a relative frequency of 6.0%. Screw perforations were observed in 3.3%, and implant dislocations occurred in 3.0%. Less frequent were HSA or RSA dislocations (1%), nonunions (0.8%), postoperative joint infections (0.7%), and postoperative nerve damage (0.2%).

Procedural complication analysis revealed the following complication rates in descending order: plate osteosynthesis (15.6%), HSA (11.2%), intramedullary nail (11.2%), screw osteosynthesis (10.3%), double plate osteosynthesis (8.3%), and RSA (6.4%).

The most common complications for plate osteosynthesis were secondary dislocations or reduction losses (7.2%) and screw perforations (5.1%). Regarding nail osteosynthesis, these were implant dislocations (4.2%), and secondary dislocations (4.6%). Arthroplasty-specific complications were dislocations in 6.4% of HSA and 6.7% of reversed shoulder arthroplasties. Secondary dislocations of the tubercles were observed in four patients (3.4%) with the hemiarthroplasty. In screw osteosynthesis, secondary dislocations occurred in 10.3% (*n* = 4) of the patients, and one screw perforation occurred in double plate osteosynthesis.

### Operation procedure-independent revisions

Humeral head necrosis occurred in 2.7% of the patients. Peri-implant fractures and fractures caused by a new trauma (1.3%) were differentiated from periprosthetic fractures (0.4%).

### Reoperations

A total of 261 patients had another operation. In 191 patients, a reoperation was performed within 12 months of primary care (Table 4).

**Table 4:** categorization of reoperations in terms of time and procedure

Group	Reasons for reoperation	*N*	Proportion of all reoperations (*N* = 261)
1	Multiple early operations with material change and/or removal	35	13.4%
2	Multiple early operations without removal of material/with partial removal of material/with reosteosynthesis	4	1.5%
3	An early operation with complete change of procedure	37	14.2%
4	An early operation with partial material change (e.g. screws)/partial/complete removal of material	108	41.4%
5	Multiple late follow-up operations with partial or complete removal of material and/or partial removal of material (e.g. screws) or later implant change	5	1.9%
6	Late material removal with arthrolysis	42	16.1%
7	Late material removal without arthrolysis	30	11.5%
	Total	261	100.0%

Early surgery is within 12 months after primary surgery, late follow-up surgery is 12 months or longer after primary surgery

Single revisions were the most frequent procedures within 12 months after primary surgery with partial or complete removal of material (41.4%). Multiple reoperations (groups 1, 2, and 5) were performed in approximately 17% of the patients. Removal of material (groups 6 and 7) more than 12 months after implantation with partial arthrolysis accounted for 27.6% of all reoperations.

### Risk factors for complications

**Fig. 2:**
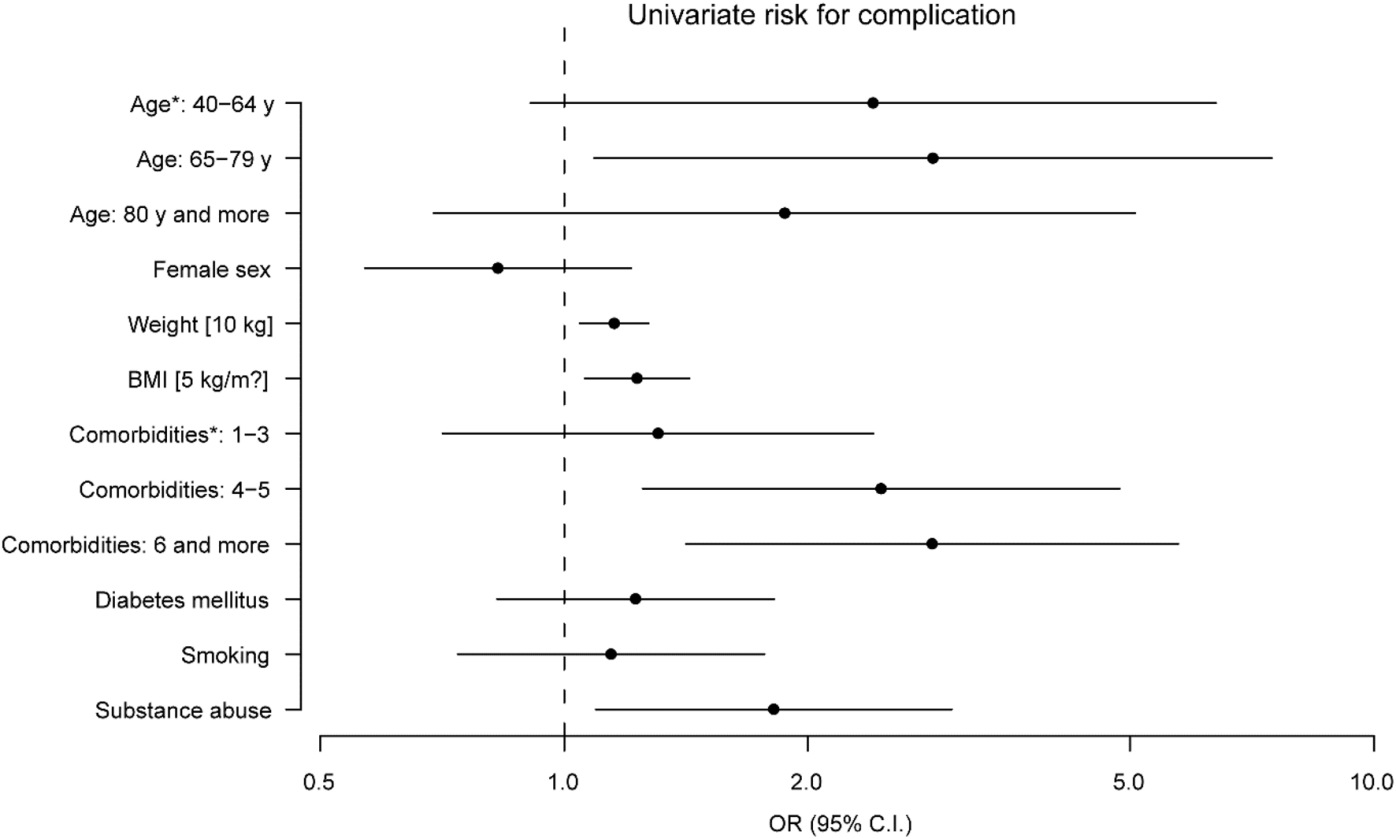
Error bar plot of possible risk factors for complications. ^a^Age group younger than 40 years, number of comorbidities: none

The groups with and without postoperative complications differed significantly in terms of the number of pre-existing conditions, body mass index (BMI), weight, and substance abuse (Table 5). Patients with complications had a higher BMI and had more comorbidities. In the bilateral logistic regression analysis, we found a significant correlation with patient age (reference category  < 40 years). The highest risk for complications occurred for the age category 65–79 years [OR 2.85 (95% confidence interval 1.09–7.48), *p* = 0.029], while in the categories 40–64 years [OR 2.41 (0.91–6.39), *p* = 0.12] and  > 80 years [OR 1.87 (0.69–5.08), *p* = 0.31] the risk was decreased. A correlation was also found for an increasing number of pre-existing conditions [one to three: OR 1.31 (0.71–2.41), *p* = 0.38], four to five: OR 2.46 (1.25–4.85), *p* = 0.008, six or more: OR 2.85 (1.41–5.74) (*p* = 0.003), substance abuse [OR 1.82 (1.09–3.01), *p* = 0.04], body weight [OR 1.15 (1.05–1.27), *p* = 0.021], and BMI [OR 1.23 (1.06–1.43), *p* = 0.022] (Fig. 2). A comparison of the surgical procedures with one another revealed that the intramedullary nail [OR 0.67 (0.42–1.07), *p* = 0.087], hemiarthroplasty [OR 0.73 (0.39–1.37)], *p* = 0.31], and RSA [OR 0.39 (0.13–1.12), *p* = 0.074] had lower risks of complications than did plate osteosynthesis, though without significance.

**Table 5:** Group comparison complication yes/no

	*N*	Complication		None		Total		*p*
		150		959		1109		Adjusted
Comorbidities	None	14	9.3%	143	14.9%	157	14.2%	< 0.001
	1–3	73	48.7%	571	59.5%	644	58.1%	
	4–5	34	22.7%	141	14.7%	175	15.8%	
	≥ 6	29	19.3%	104	10.8%	133	12.0%	
Diabetes mellitus	Yes	42	28.0%	231	24.1%	273	24.6%	0.55
	No	108	72.0%	728	75.9%	836	75.4%	
Immunosuppressive	Yes	6	4.0%	34	3.5%	40	3,6%	0.77
	No	144	96.0%	925	96.5%	1,069	96.4%	
Smoking	Yes	31	20.7%	178	18.6%	209	18.8%	0.54
	No	119	79.3%	781	81.4%	900	81.2%	
Living situation	Self-sustaining	126	84.0%	793	82.7%	919	82.9%	0.49
	Supervised	15	10.0%	81	8.4%	96	8.7%	
	Nursing home	9	6.0%	85	8.9%	94	8.5%	
Substance abuse	Yes	23	15.3%	87	9.1%	110	9.9%	0.038
	No	127	84.7%	872	90.9%	999	90.1%	
Sex	Male	48	32.2%	269	28%	317	28.6%	0.49
	Female	101	67.8%	691	72%	792	71.4%	
		Mean	SD	Mean	SD	Mean	SD	
Age		67, 1	16.8	68, 0	13, 8	67,2	16,4	0.48
Weight		73, 7	16.4	78, 2	19, 2	74,3	16,8	0.040
BMI		26, 6	5.3	28, 0	6, 5	26,8	5,5	0.037

*SD* standard deviation, *BMI* body mass index

We searched for a model to predict postoperative complications and identified one that included the following risk factors: body weight, substance abuse, age (> 40 y vs. <  = 40y), and comorbidities (three and more vs. two or less). The model, including ORs and 95% confidence intervals, is presented in Table 5. We performed a ROC analysis using the score that resulted from the model and found that the AUC = 0.64 (0.59, 0.69). The use of a threshold, a maximal sum of the sensitivity and specificity (Youden index), achieved a sensitivity is 48% and a specificity is 74%.

**Fig. 3:**
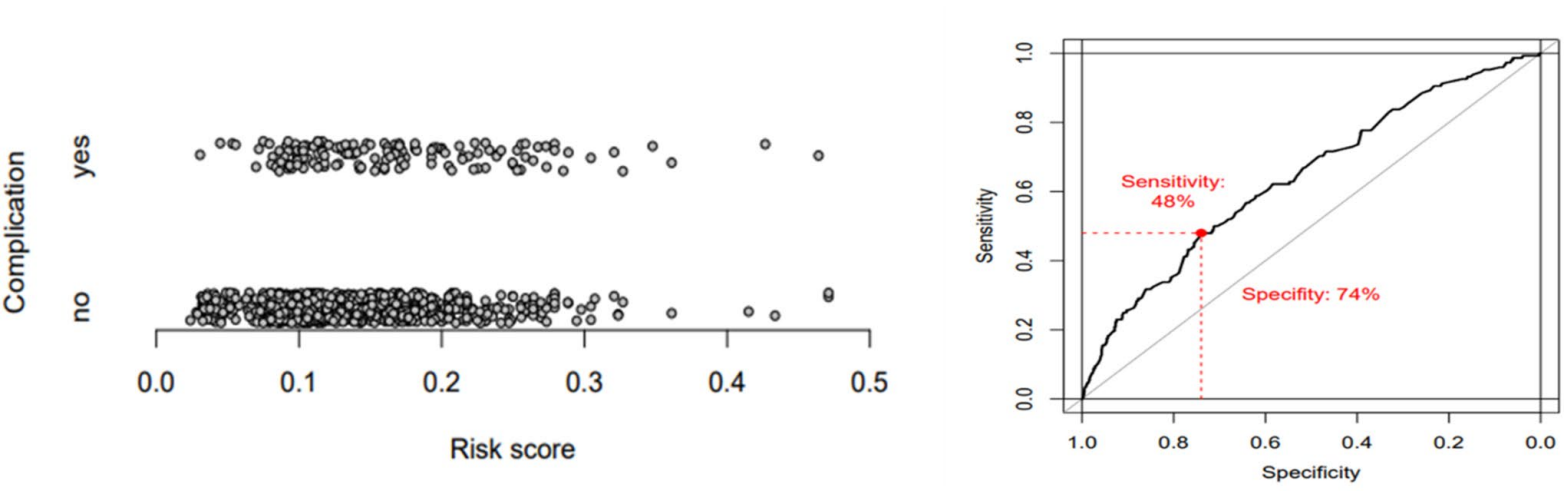
Predicted probabilities for complication and ROC curve for prediction. *ROC* receiver operating characteristic curve

Figure 3 provides a visual representation of the estimated probability of postoperative complications for the model variables of body weight, substance abuse, age (> 40y vs. <  = 40y), and comorbidities (three and more comorbidities vs. two or less).

## Discussion

Our aim was not only to identify possible risk factors but also to evaluate and weigh them to obtain a predictive value. To date, there have been no published studies that have estimated a probability of postoperative complications for identified risk factors. In our series, patients with proximal humeral fractures were characterized as follows: 75% women, over 65 years old, 82% self-sufficient, and with accidents occurring in 77% in the context of low-energy trauma. There were four or more comorbidities in almost 30% of the patients. These basic epidemiological findings are consistent with the results of other studies [6, 8, 9, 15, 17].

In our study, 13.5% of the patients experienced postoperative complications. Südkamp et al. recorded a complication rate of 34% in their prospective observational study, with 21.9% directly related to surgery and 19% that required surgical revision [8]. Katthagen et al. retrospectively reported a complication rate of 15.6%, and Rangan et al. reported a complication rate of 24% in the surgical treatment arm of a prospective randomized study [6, 18]. This heterogeneity between our results and these studies was mainly due to study-related differences in study designs, follow-up intervals, and examined implants.

In terms of surgical procedures, the lowest complication rate in our group was 6.4%, which was related to RSA. Furthermore, the complication rate for plate osteosynthesis was 15.6% compared with 11.7% for intramedullary nail osteosynthesis. Compared to other studies, similar complication rates were found for plate osteosynthesis (16–18%) and intramedullary nails (11.5–42%), while the rate was dependent on the nail design. Similar to our findings, there were higher complication rates for HSA (19.2%) compared with RSA (3.2%) [10–12]. Compared to nail or plate osteosynthesis, RSA had a collectively lower risk of complications. This was confirmed by a recent meta-analysis. [19]

The influence of epidemiological factors on the occurrence of complications has received little attention to date. Other studies mostly recorded epidemiological parameters, however, they did not investigate their relationship to complications. [14, 15, 17] Our data suggested that age was an independent risk factor and that the highest risk was for the age group of 65–79 years. To date, it has been postulated that increasing age and poor bone quality have an influence on the occurrence of a fracture [20–22]. Age may be associated with reduced bone quality; however, age is not the cause of the fracture.

Furthermore, we showed that the number of comorbidities as an indirect indication of the morbidity of the patient had an increased risk of complications. Comparable findings have been demonstrated in other studies. However, these studies did not provide any ranking of these risk factors [23–25]. The fracture-causing event in the majority of cases is low-energy trauma. Here we believe that the patient's multimorbidity is the influencing factor for recurrent falls.

Furthermore, overweight and substance abuse were identified as risk factors in our study. These risk factors are already known. [26, 27]. Even though the AUC (0.64) was low, the ROC analysis showed that in the case of postoperative complications, the predictive power due to these risk factors (body weight, substance abuse, age, and comorbidities) was at a specificity of 74% and a sensitivity of 48%. However, our approach was not able to predict a complication with high certainty.

Thus, our data provided indicators that the so-called frail patient, described as elderly with many comorbidities, required increased attention regarding indications for an operation.

### Limitations

The retrospective study design allowed explorative analyses, but a possible selection bias should be considered. Despite an intensive inclusion of all available data sources, a recording of all patients with a complete treatment course was not possible. However, it can be assumed that due to the advanced age of the patients, many patients can no longer be reached or have not presented themselves for the planned follow-up visits in the outpatient clinic. A new contact of all patients to gain an exact percentage of the follow-up did not take place. This may influence the complication rate. Furthermore, in this epidemiological analysis, follow-up examinations of the patients were not performed.

## Conclusion

Comorbidities, substance abuse, weight, and age are independent risk factors for complications of operatively treated proximal humeral fractures. If these factors are present, one can predict a postoperative complication requiring surgical revision with low sensitivity and moderate specificity. Therefore, the causes of the development of postoperative complications are complex, and the individual variability is large. When surgical or non-surgical indications are determined, independent risk factors should be considered individually with a view toward an increased risk of complications and, therefore, should be taken into account in each individual indication for surgery.

## Abbreviations

AUC: Area under the curve
BMI: Body mass index
HAS: Hemi-shoulder arthroplasty
IN: Intramedullary nail
OR: Odds ratio
ROC: Receiver operation characteristic
RSA: Reversed shoulder arthroplasty
SD: Standard deviation
